# PCA-Based Matrix CFAR Detection for Radar Target

**DOI:** 10.3390/e22070756

**Published:** 2020-07-09

**Authors:** Zheng Yang, Yongqiang Cheng, Hao Wu

**Affiliations:** College of Electronic Science and Technology, National University of Defense Technology, Changsha 410073, China; yangzheng18@nudt.edu.cn (Z.Y.); wuhao13c@nudt.edu.cn (H.W.)

**Keywords:** target detection, matrix CFAR detection, HPD matrix manifold, information redundancy reduction, PCA

## Abstract

In radar target detection, constant false alarm rate (CFAR), which stands for the adaptive threshold adjustment with variation of clutter to maintain the constant probability of false alarm during the detection, plays an important role. Matrix CFAR detection performed on the manifold of Hermitian positive-definite (HPD) covariance matrices is an efficient detection method that is based on information geometry. However, the HPD covariance matrix, which is constructed by a small bunch of pulses, describes the correlations among received data and suffers from severe information redundancy that limits the improvement of detection performance. This paper proposes a Principal Component Analysis (PCA) based matrix CFAR detection method for dealing with the point target detection problems in clutter. The proposed method can not only reduce dimensionality of HPD covariance matrix, but also reduce the redundant information and enhance the distinguishability between target and clutter. We first apply PCA to the cell under test, and construct a transformation matrix to map higher-dimensional matrix space to a lower-dimensional matrix space. Subsequently, the corresponding detection statistics and detection decision on matrix manifold are derived. Meanwhile, the corresponding signal-to-clutter ratio (SCR) is improved. Finally, the simulation experiment and real sea clutter data experiment show that the proposed method can achieve a better detection performance.

## 1. Introduction

**Figure 1:**
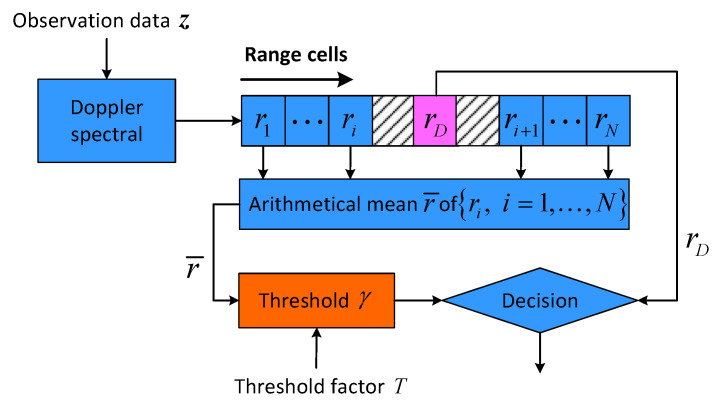
Classical FFT-CFAR detector.

Target detection in clutter environment is an important and fundamental problem in radar signal processing [1,2]. For moving target detection using coherent radar, the classical processing method is cell averaging constant false alarm rate (CA-CFAR) detection that is based on Doppler spectral estimation [3], which is also called FFT-CFAR detection, as illustrated in Figure 1. The doppler spectral $r_{i}\left(i=1,\ldots ,N\right)$ of observation data $z={\left[z_{1},z_{2},\ldots ,z_{n}\right]}^{T}$ in each range cell is estimated by FFT, and the detection decision is made by comparing $r_{D}$ with the adaptive detection threshold γ. However, for a smaller bunch of pulses, classical FFT-CFAR detection procedures are not efficient due to the low Doppler resolution and energy spread of the Doppler filter banks [4], which results in serious performance degradation [5].

**Figure 2:**
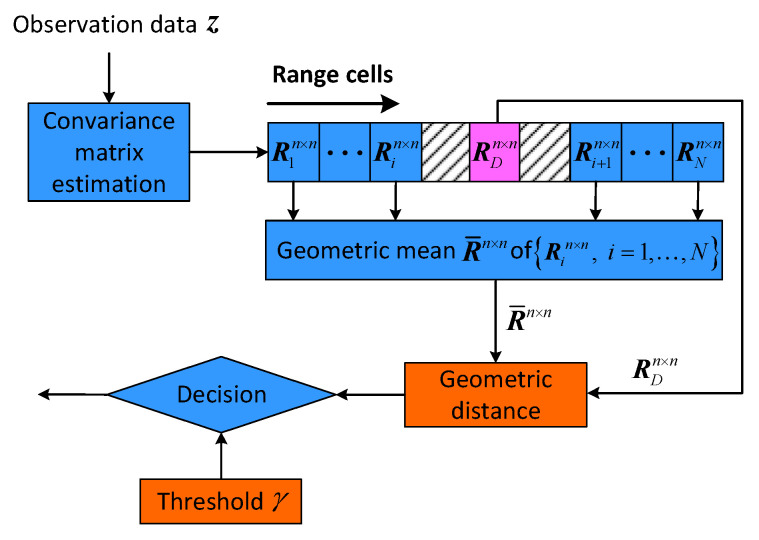
Matrix CFAR detector.

In order to overcome low resolution and energy spread issues, a new detection technique based on information geometry, which is called matrix CFAR detection, has been proposed to avoid the drawbacks of classical FFT-CFAR detection with a small bunch of pulses [6,7]. Because the covariance matrix captures the correlation information among received data, the data z in each range cell are constructed as a HPD covariance matrix, and the geometric mean and geometric distance can be calculated on a Riemannian manifold of Hermitian positive-definite (HPD) matrices [8]. HPD covariance matrice $R_{i}^{n\times n}$ is estimated by received observation data, and then geometric mean matrix ${\bar{R}}^{n\times n}$ is computed by the neighboring matrices of the cell under test, and a geometric distance is built between mean matrix ${\bar{R}}^{n\times n}$ and the cell under test $R_{D}^{n\times n}$, as illustrated in Figure 2. Finally, the matrix CFAR detection decision is made by comparing geometric distance with threshold.

**Figure 3:**
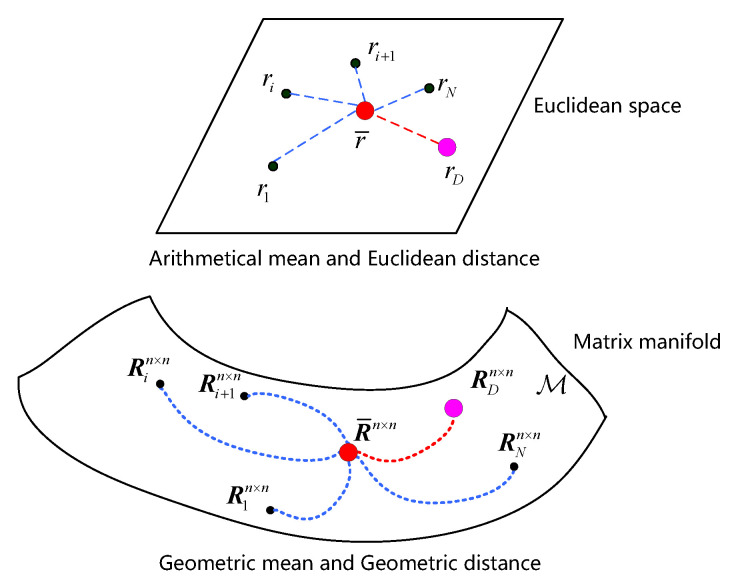
Geometrical interpretation of classical FFT-CFAR detector and matrix constant false alarm rate (CFAR) detector.

The main difference between matrix CFAR detector and classical FFT-CFAR detector is that, instead of directly using the data in Euclidean space, the HPD covariance matrices are constructed as geometric manifold [8], where the intrinsic properties of matrix that contain the information of signal can be properly exploited. A geometrical interpretation of classical detection and geometric detection [9], which perform in Euclidean space and geometric manifold, respectively, is described in Figure 3. Meanwhile, it has been verified that the matrix CFAR detector performs better than classical FFT-CFAR detector [9]. Recently, matrix CFAR detection methods are further investigated [10,11].

In practice, the elements of the HPD covariance matrix are not statistically independent, which means that the information included in the covariance matrix is highly redundant. The presence of redundant information will increase interference components when to distinguish target from clutter background, which limits the improvement of detection performance. Hence, it is necessary to extract main components and reduce redundant information of matrix to obtain better detection performance. In particular, in matrix CFAR detection, the correlations of both target and clutter data, which are caught by covariance matrix, are related to the decomposed components of matrix, and clutter components usually have weak correlations because of its randomness. It is useful to transform the data, such that the distinguishbility between the targets and the background can be maximized. Principal Component Analysis (PCA) is a widely used technique in data analysis and dimensionality reduction [12,13]. As we know, PCA for dimensionality reduction can preserve the larger eigenvalues that represent the main components and reduce the redundant information. Moreover, our goal for dimensionality reduction is to preserve the main components that mainly represent the target signal [14], thus the SCR can be improved. In comparison to other classification methods, e.g., SVM mainly works with vector data and needs to training and testing, the proposed PCA method in this paper can directly work with the HPD matrix to extract the main components and improve the SCR, which are beneficial for target detection.

Motivited by this, in this paper, PCA method, which can not only reduce the dimensionality of the Toeplitz HPD covariance matrix, but also capture the main components and reduce the redundant information, is carried out to remove the weakly correlated redundant components with smaller eigenvalues, thus the distinguishbility between target and clutter can be enhanced. Here, empirical mean (EM) algorithm and regularized Burg (RB) algorithm are considered to estimate the covariance matrix, respectively. Subsequently, a transformation matrix (or mapping matrix), which can map the higher-dimensional matrix space to a lower-dimensional matrix space, is constructed. Thus, a PCA-based matrix CFAR detector is proposed, and the improvement of signal-to-clutter ratio (SCR) is analyzed. Moreover, the detection statistics and detection decision are calculated by the lower-dimensional covariance matrices. Finally, simulation experiment and real sea clutter data experiment demonstrate the proposed PCA-based matrix CFAR detection method can achieve a better detection performance.

The remainder of this paper is organized as follows. Section 2 introduces PCA method for information redundancy reduction of covariance matrix and analyses the detection performance. Subsequently, Riemannian geometric metrics and means on HPD matrix manifold are presented in Section 3. The proposed PCA-based matrix CFAR detector is derived in Section 4. Section 5 shows the detection performance results obtained from simulated data and real sea clutter data. Finally, a conclusion is drawn in Section 6.

## 2. PCA for Information Redundancy Reduction of Covariance Matrix

In this section, we mainly present the PCA method for information redundancy reduction of covariance matrix, and then a dimensionality and redundant information reduced matrix is obtained. Finally, an analysis is given on the detection performance after PCA.

### 2.1. PCA Method for Covariance Matrix

The radar received observation pulse data for range cell has a complex vector form $z={\left[z_{1},z_{2},\ldots ,z_{n}\right]}^{T}$, where *n* is the length [8]. Assuming there exists a target, whose behavior on reflectivity or speed is different from its environment, then the observed vector

(1) z=s+c,

where $s={\left[s_{1},s_{2},\cdots ,s_{n}\right]}^{T}$ is a complex target signal vector and $c={\left[c_{1},c_{2},\cdots ,c_{n}\right]}^{T}$ is a complex environmental clutter vector. For Equation (1), assuming s and c are independent, the corresponding covariance matrix of the observed vector can be described as

(2) $$\begin{array}{cc} R_{z} & =E\left[zz^{H}\right]\\ \; & =E\left[\left(s+c\right){\left(s+c\right)}^{H}\right]\\ \; & =E\left[ss^{H}\right]+E\left[cc^{H}\right]\\ \; & =R_{s}+R_{c}, \end{array}$$

where E denotes statistical expectation, ${\left(\cdot \right)}^{H}$ denotes the conjugate transpose operator, and $R_{s}$ and $R_{c}$ represent the covariance matrix of the target signal vector and clutter vector, respectively. Supposing that $R_{z}$ is symmetric matrix, then we apply eigenvalue decomposition (EVD) to $R_{z}$, which can be written as

(3) $$\begin{array}{cc} R_{z} & =V_{z}\Sigma_{z}V_{z}^{H}\\ \; & =V_{s}\Sigma_{s}V_{s}+V_{c}\Sigma_{c}V_{c}, \end{array}$$

where eigenvector matrix $V_{z}=\left[V_{s},V_{c}\right]$, $V_{s}$ and $V_{c}$ are eigenvector matrices that correspond to larger eigenvalues and smaller eigenvalues, respectively. Basically, target signal components have relatively larger eigenvalues. Hence, the target signal space is spanned by $V_{s}$ and the clutter space is spanned by $V_{c}$. $\Sigma_{z}$ is known as eigenvalue matrix of $R_{z}$, which is expressed as

(4) $$\Sigma_{z}=\left[\begin{array}{cc} \Sigma_{s} & \\ & \Sigma_{c} \end{array}\right]=\left[\begin{array}{ccccc} \lambda_{1} & & & & \\ & \ddots & & & \\ & & \lambda_{p} & & \\ & & & \ddots & \\ & & & & \lambda_{n} \end{array}\right],$$

where $\lambda_{1}\geq \cdots \geq \lambda_{p}\geq \cdots \geq \lambda_{n}$ and $\Sigma_{s}=diag\left(\lambda_{1},\lambda_{2},\cdots ,\lambda_{p}\right)$ contains *p* larger eigenvalues, $\Sigma_{c}=diag\left(\lambda_{p+1},\lambda_{p+2},\cdots ,\lambda_{n}\right)$ contains (n−p) smaller eigenvalues represent the redundant information.

Because the criteria of proposed PCA method are to preserve *p* larger eigenvalues, and these eigenvalues, which are related to the distribution of signal power in the spectrum, represent the main power of target signal, then based on the same length for signal and spectrum, the best order *p* is the number of point for the concentrated distribution of power in the spectrum.

Hence, based on matrix EVD, the PCA method is performed. Firstly, we preserve the *p* larger eigenvalues that represent the target signal space, which means the redundant information can be reduced. Secondly, the corresponding eigenvector matrix $V=\left[v_{1},v_{2},\cdots ,v_{p}\right]$, which is also called transformation matrix, is constructed, where v is eigenvector and $V^{H}V=I$. Finally, the received observation pulse data can be expressed as

(5) $$x=V^{H}z.$$

Subsequently, by applying the PCA method, a dimensionality and redundant information reduced covariance matrix is given by

(6) $$\begin{array}{cc} R_{x} & =E\left[xx^{H}\right]=E\left[V^{H}zz^{H}V\right]\\ \; & =V^{H}R_{z}{V=\Sigma }_{z}^{↓}, \end{array}$$

where E denotes statistical expectation, the notation ↓ denotes dimensionality reduction operation. $\Sigma_{z}^{↓}=diag\left(\lambda_{1},\lambda_{2},\cdots ,\lambda_{p}\right)$ is dimensionality-reduced eigenvalue matrix, and the quantitative result of information redundancy is described as $\sum_{i=p+1}^{n}\lambda_{i}$.

### 2.2. Analysis of Detection Performance

Generally, SCR limits detection performance. Thus, we focus on deriving a concise analysis for SCR before and after PCA. Assuming that clutter is stationary and independent with average power $\sigma^{2}$, the EVD of covariance matrix $R_{z}$ is given by

(7) $$R_{z}=\sum_{i=1}^{p}(\lambda_{i}+\sigma^{2})v_{i}{v_{i}}^{H}+\sum_{i=p+1}^{n}\sigma^{2}v_{i}{v_{i}}^{H},$$

where $\lambda_{i}$ and $v_{i}$ are corresponding eigenvalue and eigenvector, respectively. SCR${}_{1}$$=\sum_{i=1}^{p}\lambda_{i}/\;\sum_{i=1}^{n}\sigma^{2}$. Based on Equation (4), PCA is employed to the covariance matrix of radar observation data, by removing the smaller eigenvalues that mainly represent the redundant information and preserving the larger eigenvalues mainly represent the target signal components, then SCR${}_{2}$$=\sum_{i=1}^{p}\lambda_{i}/\;\sum_{i=1}^{p}\sigma^{2}$. Obviously, SCR${}_{1}$ < SCR${}_{2}$. Thus, PCA for covariance matrix can lead to the improvement of SCR.

## 3. Geometric Metrics on HPD Matrix Manifold

This section is devoted to introducing the geometry space of HPD matrix, which we call Riemannian manifold or HPD matrix manifold. A Toeplitz HPD Covariance Matrix, whose set forms a differentiable Riemannian manifold, is mapped from observation data [15,16]. Subsequently, the geometric metrics, such as Riemannian distance (RD) and Kullback–Leibler divergence (KLD) on HPD matrix manifold, are presented [16,17].

### 3.1. Mapping from Observation Data to HPD Matrix Manifold

As described in Section 2, the received observation data in a range cell is a complex vector $z={\left[z_{1},z_{2},\ldots ,z_{n}\right]}^{T}$, which is assumed as a complex circular multivariate Gaussian distribution. Then, we transform the received data into covariance matrices according to the correlation coefficients. Thus, the covariance matrix, which is Toeplitz in the case of a locally stationary time series, can be given as [8,18]

(8) $$\begin{array}{c} A_{z}=E\left[zz^{H}\right]=\left[\begin{array}{cccc} a_{0} & {\bar{a}}_{1} & \cdots & {\bar{a}}_{n-1}\\ a_{1} & a_{0} & \cdots & {\bar{a}}_{n-2}\\ \vdots & \ddots & \ddots & \vdots \\ a_{n-1} & a_{n-2} & \cdots & a_{0} \end{array}\right],\\ a_{k}=E\left[z_{i}{\bar{z}}_{i+k}\right],\;\;\;\;\;0\leq k\leq n-1,\;\;\;0\;\leq i\leq n-1, \end{array}$$

where ${\left(\cdot \right)}^{H}$ denotes the conjugate transpose operator, $\bar{z}$ denotes the complex conjugate of *z*, E denotes statistical expectation, and $a_{k}=E\left[z_{i}{\bar{z}}_{i+k}\right]$ is called the correlation coefficient [8]. $A_{z}$ is strictly positive definite with $A_{z}=A_{z}^{H}$. Statistical covariance matrix is difficult to obtain because of the limited number of received sample data. Therefore, we consider two estimation algorithms:

(i) Empirical mean algorithm: the ergodicity of a wide sense stationary is utilized, and the correlation coefficient $a_{k}$ can be calculated by averaging over time

(9) $${\hat{a}}_{k}=\frac{1}{n-k}\sum_{j=0}^{n-1-k}z_{j}{\bar{z}}_{j+k}\;\;\;\;\;,\;\;\;\;\;0\leq k\leq n-1.$$

(ii) Regularized Burg algorithm: the reflection coefficient of the radar observation data is estimated to construct the Toeplitz HPD covariance matrix [8,19].

Hence, let C(n) be the set of vectors of observation data with *n* variables and P(n) be the set of corresponding complex HPD matrices with order *n*. Subsequently, P(n) can be equipped with certain Riemannian structure through the mapping [5]:

(10) $$\psi :C\left(n\right)\to P\left(n\right),\begin{array}{cc} & z \end{array}\to A\in P\left(n\right),$$

where P(n) forms a differentiable Riemannian manifold with non-positive curvature [20], and a HPD matrix manifold is a closed, self-dual convex cone, and it serves as a canonical high dimension symmetric geometry space, whose size grows as $n^{2}$ [21].

### 3.2. Geometric Metrics on Matrix Manifold

Let $H\left(n\right)=\left\{A,A^{H}=A\right\}$ denotes the space of all n×n Hermitian matrices. For A∈H(n), if the quadratic form $x^{H}Ax>0,\forall x\in C\left(n\right)$, then A>0. Hence, as stated above, the elements of P(n) form an open subset of H(n) and it can be equipped with a Riemannian structure. The relation between H(n) and P(n) is given as [17]

(11) P(n)={A∈H(n),A>0}.

(i)Riemannianmetric: A Riemannian metric [17,20], which is also called geodesic distance or Riemannian distance, is an inner product

(12) $${\langle X,Y\rangle }_{A}=tr\left(A^{-1}XA^{-1}Y\right),$$

where A∈P(n) and X,Y∈P(n), and $\langle X,Y\rangle =\langle Y,X\rangle$. In particular, the corresponding norm is $∥X∥={\langle X,X\rangle }^{1/2}$ and $\langle X,Y\rangle =tr\left(XY^{H}\right)$.

It is known that P(n) is a differentiable manifold, for $A_{n}\in P\left(n\right)$, the infinitesimal arclength

(13) $$ds={∥{A_{n}}^{-1/2}dA_{n}{A_{n}}^{-1/2}∥}_{F}$$

defines a Riemannian metric on P(n). In order to measure the distance between two points $A_{1},A_{2}\in P\left(n\right)$, by integrating (13), Riemannian distance (RD) is given as

(14) $$\begin{array}{cc} d_{R}^{2}\left(A_{1},A_{2}\right) & ={∥\log({A_{1}}^{-1/2}A_{2}{A_{1}}^{-1/2})∥}_{F}^{2}\\ & =\sum_{k=1}^{N}\log^{2}\left(\lambda_{k}\right), \end{array}$$

where $\lambda_{k}$ is the *k*th eigenvalue of ${A_{1}}^{-1/2}A_{2}{A_{1}}^{-1/2}$, $\log\left(\cdot \right)$ is the log map on the HPD matrix manifold P(n). This metric is a particular case of intrinsic metric in Siegel Upper-half space invariant under the automorphisms.

(ii)KLdivergence: On matrix manifold, KL divergence (KLD) is another metric besides geodesic distance. The similarity between two probability distributions is measured by KL divergence, which is termed the relative entropy in information theory [9]. However, KL divergence has all of the properties of a distance function, but it does not satisfy the triangle inequality [17]. Additionally, it plays an important role in the theory of statistical inference [22].

Hence, based on HPD matrix manifold, the KL divergence between two covariance matrices can be defined as

(15) $$d_{KL}\left(A_{1},A_{2}\right)=tr\left({A_{2}}^{-1}A_{1}-I\right)-\log\left|{A_{2}}^{-1}A_{1}\right|.$$

## 4. PCA-Based Matrix CFAR Detector

**Figure 4:**
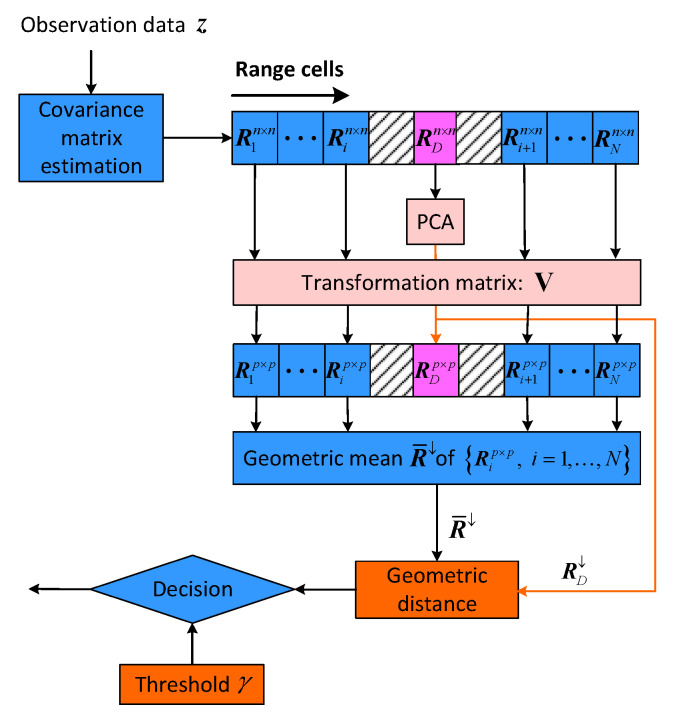
Principal Component Analysis (PCA)-based matrix CFAR detector.

It is known that matrix CFAR detector is a geometric detector that performs on the matrix manifold [9], in which the intrinsic properties of the covariance matrix can be exploited and utilized [5]. According to the PCA method for covariance matrix that have been mentioned in Section 2, we devise a PCA-based matrix CFAR detector, as illustrated in Figure 4.

To be more concrete, first, the observation pulses data $z={\left[z_{1},z_{2},\ldots ,z_{n}\right]}^{T}$ in each range cell is estimated as a HPD covariance matrix based on EM algorithm or RB algorithm. After that, the PCA method is applied to the HPD covariance matrix $R_{D}$ of the cell under test with order *n*. Subsequently, the *p*(p<n) larger eigenvalues are preserved as $\Sigma_{D}^{↓}=diag\left(\lambda_{1},\lambda_{2},\cdots ,\lambda_{p}\right)$, herein

(16) $$R_{D}^{↓}=\Sigma_{D}^{↓},$$

where $R_{D}^{↓}$ is p×p dimensionality-reduced covariance matrix of the cell under test and the quantity of redundant information is $\sum_{i=p+1}^{n}\lambda_{i}$. Meanwhile, the transformation matrix is constructed as

(17) $$V=\left[v_{1},v_{2},\cdots ,v_{p}\right],$$

which is also called mapping matrix. Subsequently, the neighboring covariance matrices $\left\{R_{1}^{n\times n},\cdots ,R_{i}^{n\times n},R_{i+1}^{n\times n},\cdots ,R_{N}^{n\times n}\right\}$ of $R_{D}^{n\times n}$ are mapped by V, as

(18) $$R^{↓}=V^{H}R^{n\times n}V,$$

where $R^{n\times n}$ is an arbitrary HPD covariance matrix that surrounds the cell under test, and $R^{↓}$ is the p×p dimensionality-reduced HPD covariance matrix. Thus, a new set of HPD covariance matrices with lower dimensionality are generated as $\{R_{1}^{↓},R_{2}^{↓},\cdots ,R_{N}^{↓}\}$.

According to the new set of dimensionality-reduced matrices, we proceed to carry out geometric signal detection processing. It is known that geometric mean has been widely investigated in many years [23,24], which is proved as existence and uniqueness for a set of HPD matrices [25].

In Euclidean space, the mean of a finite and positive set $\left\{z_{1},z_{2},\ldots ,z_{n}\right\}$ is arithmetic mean, which is given by

(19) $$\bar{z}=\frac{1}{n}\sum_{i=1}^{n}z_{i}=\arg\min_{z>0}\frac{1}{n}\sum_{i=1}^{n}\left|z-z_{i}\right|.$$

However, in HPD matrix manifold space, it is the geometric mean ${\bar{R}}^{↓}$ for the set of HPD matrices. Hence, the corresponding geometric mean of Riemannian metric can be computed as

(20) $${\bar{R}}^{↓}=\arg\min_{R^{↓}}\frac{1}{N}\sum_{i=1}^{N}d_{R}^{2}\left(R_{i}^{↓},R^{↓}\right).$$

Equation (20), which can be usually calculated by fix point algorithm, is derived as

(21) $${\bar{R}}_{t+1}^{↓}={{\bar{R}}_{t}^{↓}}^{1/2}\exp\left\{-\sigma \left(\sum_{k=1}^{N}\log({{\bar{R}}_{t}^{↓}}^{-1/2}R_{k}^{↓}{{\bar{R}}_{t}^{↓}}^{-1/2})\right)\right\}{{\bar{R}}_{t}^{↓}}^{1/2}$$

where *t* is the iteration index and σ is the step size [17].

Meanwhile, the KL mean

(22) $${\bar{R}}^{↓}=\arg\min_{R^{↓}}\frac{1}{N}\sum_{i=1}^{M}d_{KL}^{2}\left(R_{i}^{↓},R^{↓}\right)$$

can be derived as

(23) $${\bar{R}}^{↓}={\left(\frac{1}{N}\sum_{i=1}^{N}{R_{i}^{↓}}^{-1}\right)}^{-1}.$$

The geometric distance $d(R_{D}^{↓},{\bar{R}}^{↓})$ is computed to compare with the detection threshold γ to decide whether there is the target or not.

Therefore, the detection decision of PCA-based matrix CFAR detector is

(24) $$d\left(R_{D}^{↓},{\bar{R}}^{↓}\right)\overset{target}{\underset{no\;target}{≷}}\gamma ,$$

where $d(R_{D}^{↓},{\bar{R}}^{↓})$ is the detection statistics, and detection threshold γ related to the clutter power level can be determined by the desire probability false alarm $P_{fa}$. If the distance is greater than threshold, then we can conclude that there is a target at the location of the cell under test. In our work, γ is derived by Monte Carlo method based on desire constant false alarm probability. A concise procedure is described in Table 1.

Moreover, since the complexity of the inversion of matrix is $n^{3}$ (*n* is the pulse length) and the complexity of the matrix mapping is $pn^{2}$ (*p* is the dimensionality after PCA), then the computational complexity of the proposed method mainly goes from $O(Nn^{3})$ to $O(Npn^{2})$ (*N* is the number of range cell). Thus, the computational complexity will become lower with dimensionality reducing.

## 5. Numerical Experiments

In this section, simulated data and real sea clutter data are performed to evaluate the detection performance of the proposed PCA-based matrix CFAR detector for. The performance results are demonstrated in the case of detection probability under the desired false alarm probability.

### 5.1. Simulation Experiments

Here, we assume that the pulse repetition frequency is 1000 Hz, and data samples are from n=7 received short pulses train, while there are M=17 range cells and G=2 guard cells. The point target in our detection scenario is modeled as

(25) s=αp,

where α represents the target power and $p=(1,e^{j2\pi f_{D}},\ldots ,e^{j2\pi \left(n-1\right)f_{D}})$, normalized Doppler frequency $f_{D}=0.15$ Hz. This is a single frequency (SF) signal model that is adequate for a stationary or slowly moving target on the sea surface. Because K-distributed clutter is a proper model for sea clutter, then, in this study, the clutter is set as K distribution, whose amplitude pdf is given below:

(26) $$p\left(x\right)=\frac{2\mu }{\Gamma (\nu )}\left(\frac{\mu x}{2}\right)K_{\nu -1}\left(\mu x\right),$$

where ν and μ denote shape parameter and scale parameter, respectively. Γ(·) is the Gamma function, and $K_{\nu -1}\left(\cdot \right)$ is the modified Bessel function of the second kind with order ν−1. Shape parameter ν=1 and scale parameter μ=0.5.

**Figure 5:**
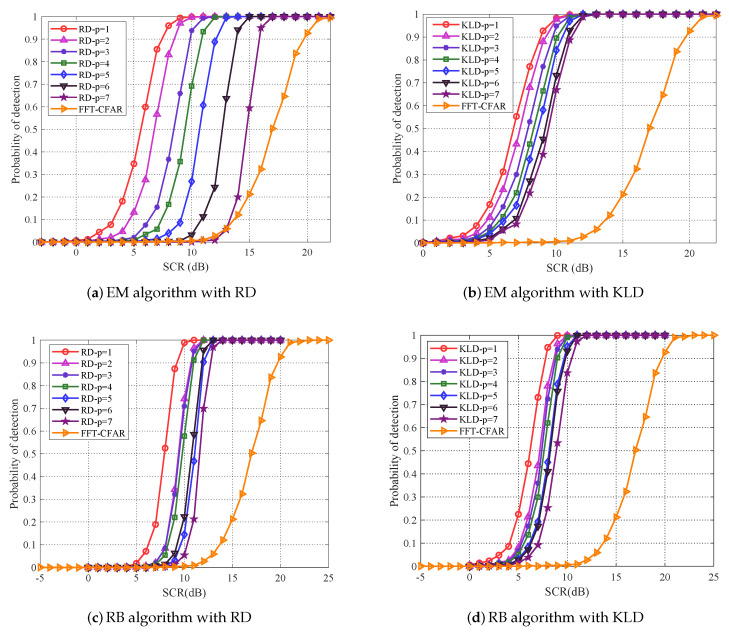
(**a**,**b**) plot the detection performance of EM algorithm, and (**c**,**d**) plot the detection performance of RB algorithm for proposed PCA method.

Firstly, the covariance matrix is estimated by the empirical mean (EM) algorithm and regularized Burg (RB) algorithm, respectively. We mainly choose Remiannian distance (RD) and KL divergence (KLD) as the geometry distance metric. The detection performance of proposed PCA method are obtained by Monte Carlo simulations, the target (25) is located at the 9th range cell, and the probability of false alarm is set as $P_{fa}=10^{-4}$. Here, SCR varies from 0 to 22 dB with an interval of 1 dB and the detection performance of p×p (p=n) covariance matrix represents the original matrix CFAR detection. As illustrated in Figure 5, just as verified, the original matrix CFAR detector (p=n) outperforms classical FFT-CFAR detector, and KLD outperforms RD. It can be noted from Figure 5 that the detection performance are gradually increasing with matrix dimensionality reducing. As we can see, based on RD, a substantial performance improvement is achieved, when p=1, the detection performance of EM algorithm has improved about 9 dB when detection probability achieves 0.8, while RB algorithm has improved about 4 dB, particularly, the EM algorithm is superior to RB algorithm about 2 dB. Similarly, based on KLD, when p=1, the detection performance of EM algorithm and RB algorithm are both improved about 2.5 dB and RB algorithm is superior to EM algorithm about 1 dB.

**Figure 6:**
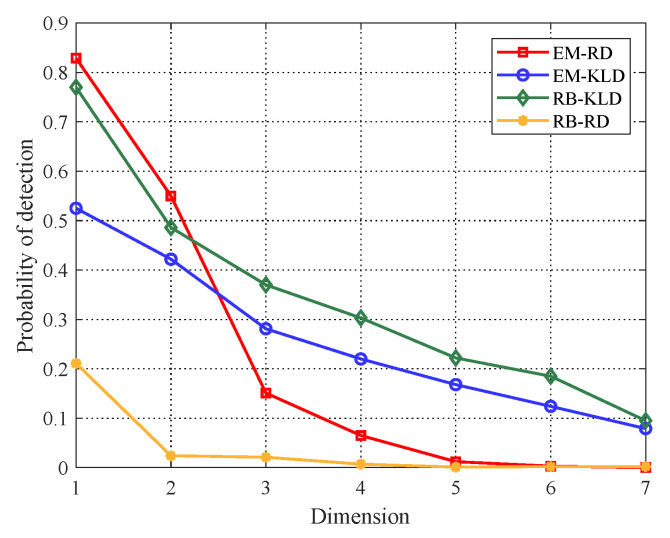
Detection probabilities of EM and RB algorithms versus dimensions, SCR = 7 dB.

Subsequently, the comparison of detection probabilities versus dimensions for the two covariance estimation algorithms are performed. Here, we assume that SCR = 7 dB. As shown in Figure 6, with matrix dimensionality reducing, the detection probabilities of both estimation algorithms are improved. Although KLD keeps the superiority, RD with EM algorithm can perform better than KLD when matrix dimension p=2 or p=1. Such results can not only prove the improvement of detection performance after PCA, but also show the possibility that RD outperforms KLD.

**Figure 7:**
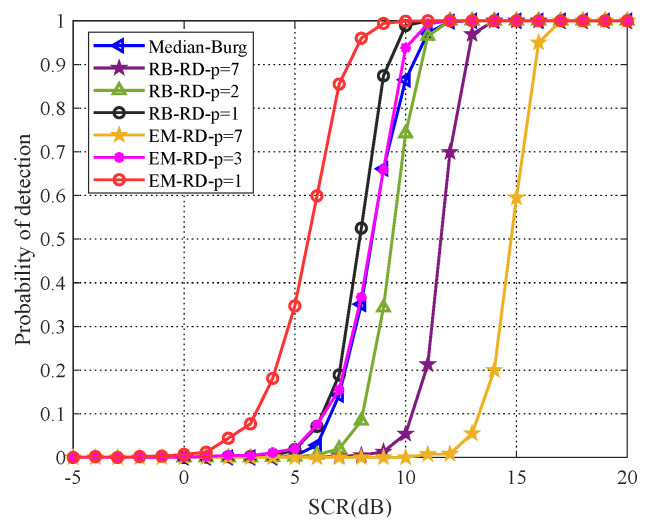
Detection performance comparison for PCA method and Median Burg method.

Moreover, it is known that Median Burg method is robust for estimating the Toeplitz covariance matrix in some cases, which has better detection performance than the very popular fixed point estimator and OS-CFAR detector for radar point target [26]. Hence, in the simulated scenario of this paper, we draw a comparison between PCA method and Median Burg method. As shown in Figure 7, the Median Burg method, which is the same as EM algorithm (p=3), outperforms both original matrix CFAR detection methods (p=7). Besides, with dimensionality reducing, PCA method has the ability to outperform Median Burg method. When p=1, the EM algorithm and RB algorithm are superior to the Median Burg method, about 3dB and 1dB, respectively.

**Figure 8:**
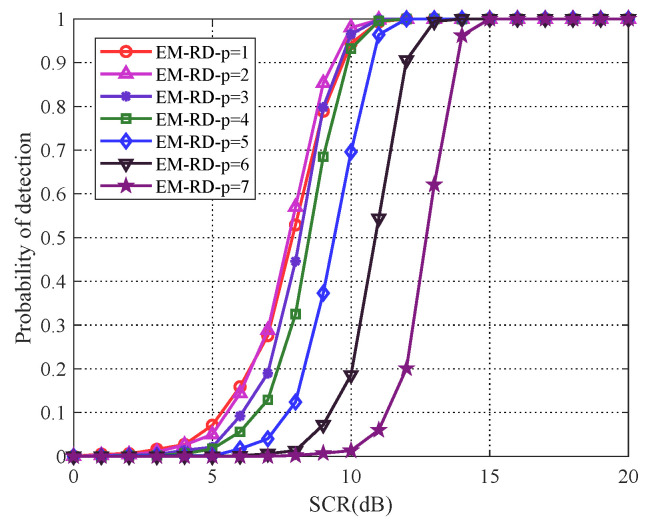
Detection performance of proposed PCA method with linear frequency modulation (LFM) signal model.

**Figure 9:**
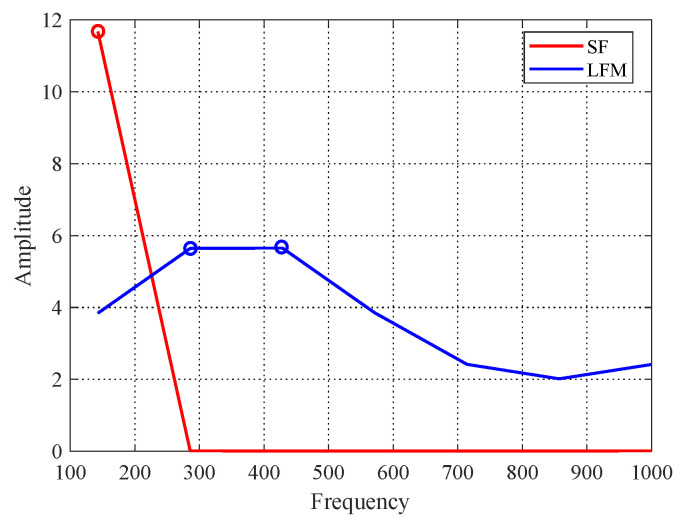
Spectrums of signal models.

In addition, because continuous waveform and frequency modulation waveform are commonly used in radar cases, then single frequency (SF) waveform (25) was chosen above, and a typical frequency modulation waveform that is linear frequency modulation (LFM) waveform is considered as another scenario to evaluate detection performance of the proposed method. The steering vector is modeled as $l=(1,e^{\left(j2\pi f_{D}+j\pi k\right)},\ldots ,e^{\left(j2\pi f_{D}+j\pi k{\left(n-1\right)}^{2}\right)})$, where *k* is the chirp rate, and the pulse repetition frequency and bandwidth are set to 1000 Hz and 500 Hz, respectively. The EM algorithm under RD is applied to the simulation. As Figure 8 shows, the detection performance of the proposed PCA method is improved with dimensionality reducing, which demonstrates that the proposed method can still achieve a better performance with respect to the different scenario. In particular, it is noteworthy that the best performance of the this scenario comes to p=2, and the scenario of SF signal model comes to p=1. The reason is that the selection of order *p* is related with the distribution of signal power in the spectrum. As exhibited in Figure 9, based on the spectrum of two signals, the number of point for the concentrated distribution of power in the spectrums are 1 (SF) and 2 (LFM), respectively, which means that the main power in the scenarios can be preserved when p=1 and p=2, respectively, so that the best performance has been obtained.

**Figure 10:**
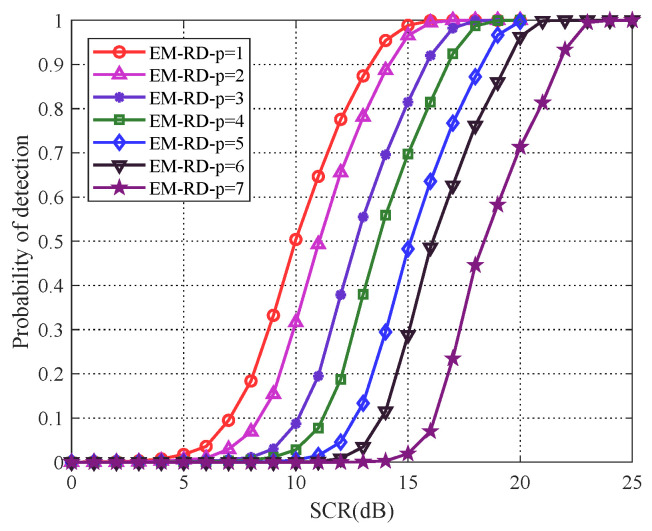
Detection performance of proposed PCA method with extended target.

Further, the extended target is used to validate the performance of proposed PCA method. For simplifying a model, we assume an extended target as a set of scattering points belong to 7 consecutive range cells, which are from the 6th range cell to the 12th range cell, and each range cell contains a useful return from target. Subsequently, the point target model (25) is employed, and EM algorithm under RD is implemented in the simulation. As illustrated in Figure 10, although the extended target results in performance degradation, an improvement still has been achieved. When p=1, it also has been improved, about 9 dB. Such significant improvement in detection performance can validate the proposed PCA method has superiority with respect to the case of extended target, and more complex situations will be considered in the future work.

### 5.2. Numerical Experiments for Real Sea Clutter Data

The real sea clutter data in the file 19980205_185111_ANTSTEP.CDF is used from the famous McMaster University IPIX radar [27]. The radar is a coherent and polarimetric X-Band pulse Doppler (PD) radar with a carrier frequency of 9.39 GHz, the azimuth beam width is $0.9^{\circ }$, and the pulses repetition frequency (PRF) is 1 KHz. Since there are four polarization modes available, the horizontal polarization (HH) is exploited in order to carry out the experiment. Meanwhile, the data contain 27 range cells, each range cell has 60,000 coherent pulses, and the range resolution is 3 m. Two preprocessing procedures, which are removing mean and standard deviation from the I and Q channels separately and removing the phase inbalance, are performed.

**Figure 11:**
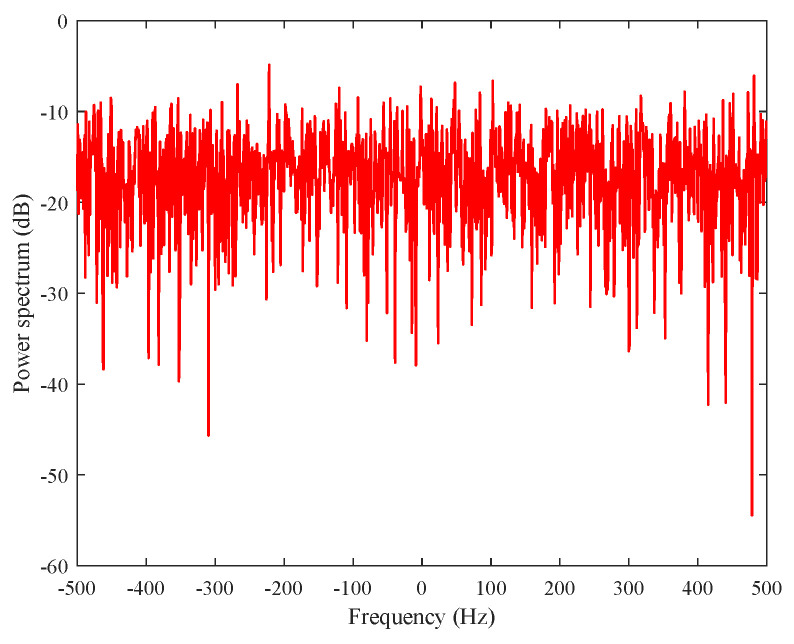
Clutter power spectrum for IPIX radar data.

In our experiments, 17 range cells with two guard cells are considered, and the length of the pulses in each range cell is set as 7. Here, the probability of false alarm is assumed as $P_{fa}=10^{-3}$, due to the limited length of clutter data. Because there is no target information in the dataset, the point target model (25) with normalized Doppler frequency $f_{D}=0.15$ Hz is added into the 9th range cell in the numerical experiments. Monte Carlo simulations are carried out in order to evaluate the detection performance. Figure 11 illustrates the clutter power spectrum of the 9th range cell, which clearly shows that the clutter background for detection contains both zero Doppler and no zero Doppler.

**Figure 12:**
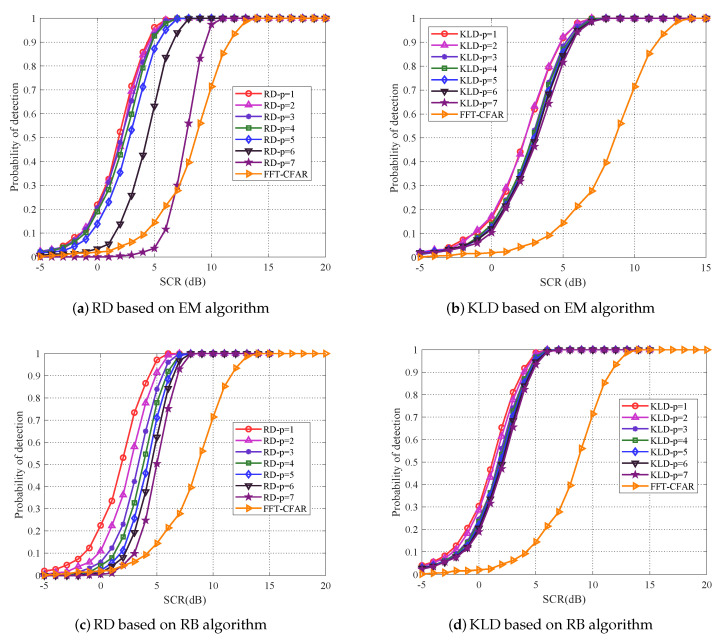
For IPIX radar data, (**a**,**b**) plot the EM algorithm and (**c**,**d**) plot the the detection performance of RB algorithm for proposed PCA method.

**Figure 13:**
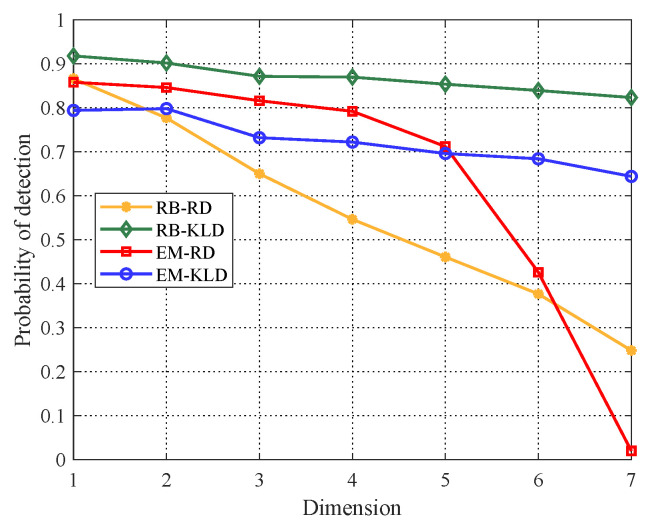
For IPIX radar data, detection probabilities of EM and RB algorithms versus dimensions, SCR = 4 dB.

The detection performance of the EM algorithm and RB algorithm for proposed PCA method are carried out using the dataset. As described in Figure 12 and Figure 13, geometric detection and KLD still maintain their superiority, and the detection performance of both algorithms have been improved with matrix dimensionality reducing. As we can see, when p=1, the RB algorithm is superior to EM algorithm about 1 dB under KLD, while they are almost the same under RD. In addition, it is worth noting that the best detection performance is also achieved by p=1, like the discussion above, in this detection scenario, the number of point for the concentrated distribution of power in the spectrum is 1.

**Figure 14:**
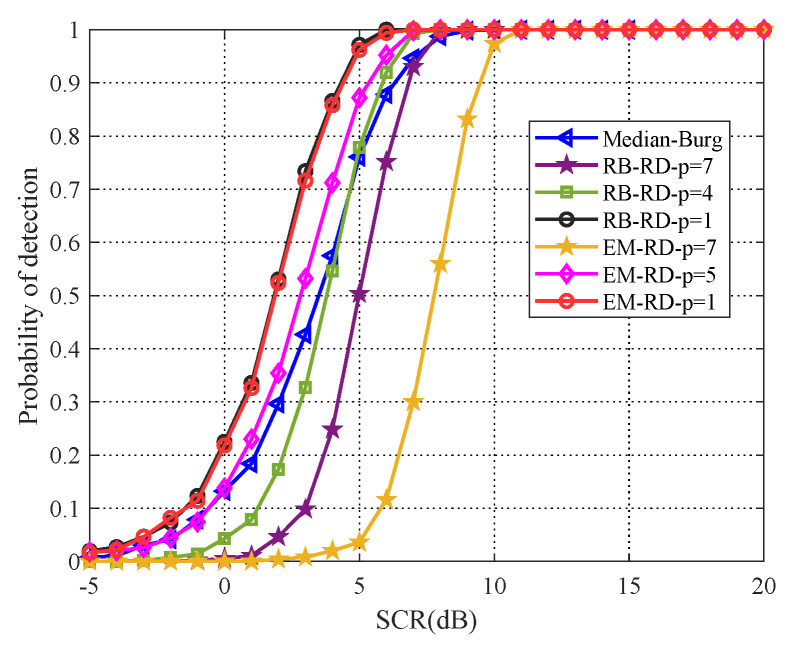
Detection performance comparison for PCA method and Median Burg method based on IPIX radar data.

Subsequently, the comparative detection performance result of the proposed method and Median Burg method in real clutter data is illustrated as Figure 14, with dimensionality reducing, it is clearly shown that PCA method outperforms the Median Burg method.

**Figure 15:**
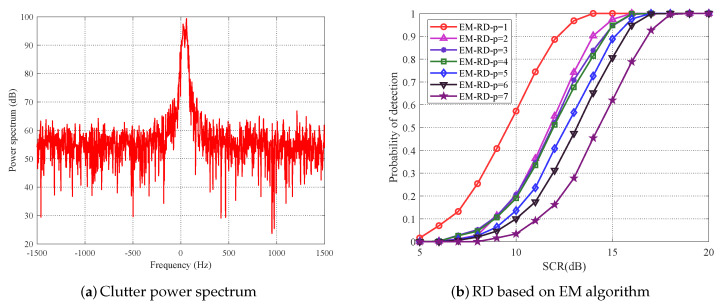
For radar data of NAU, (**a**) plots the clutter power spectrum and (**b**) plots the detection performance of proposed method.

In addition, another set of real sea clutter data (file 20191012112446_01_staring) from the practical X band solid-state power amplifier radar system is utilized via the sea-detecting X-band radar and data acquisition program of naval aeronautical university (NAU) [28]. Here, the radar with horizontal polarization (HH) that works in staring mode transmits LFM signal of pulse width 3 μs. Meanwhile, the carrier frequency is 9.3 GHz, PRF is 3 KHz, and range resolution is 6 m. Subsequently, data in the file that contain 5250 range cells and 10,000 coherent pulses in each range cell are exploited, and the target model in Equation (25) is employed. In addition, the concrete experiment parameters and detection scenario are the same as the experiments based on IPIX radar data. Thus, the corresponding clutter power spectrum of the 9th range cell is shown in Figure 15a, and the experimental result of proposed method is depicted as Figure 15b. As we can see, the improvement of detection performance demonstrates the claimed advantages of the proposed method.

Further, an intuitive detection effectiveness of PCA-based matrix CFAR detector is shown. Because RD has achieved the significant improvement, it is considered. Based on IPIX radar data, five point targets set as Table 2 are added into the corresponding range cells.

**Figure 16:**
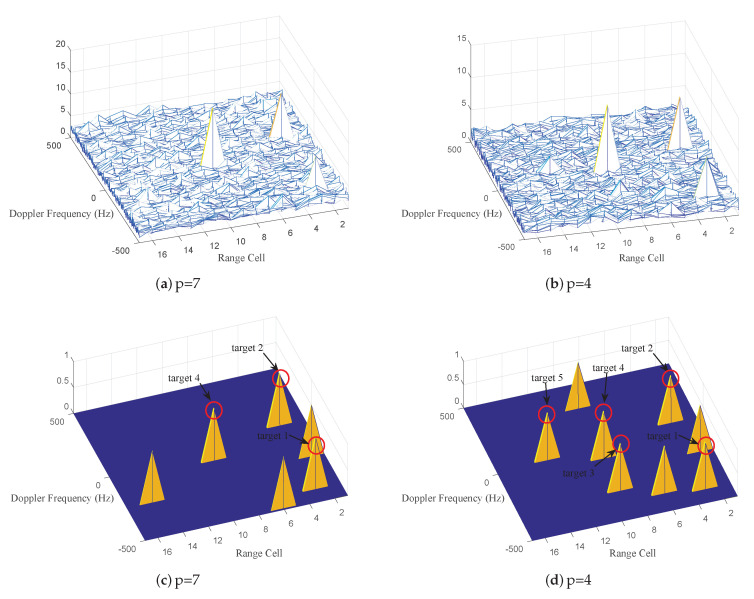
For IPIX radar data, (**a**,**b**) plot the detection statistics of each range cell with RD in Range-Doppler domain and (**c**,**d**) plot the detection results with RD under 3 false alarms.

Herein, we mainly compare the geometric detection effectiveness of original 7×7 covariance matrix with 4×4 covariance matrix in Range–Doppler domain. The detection statistics in all cells are depicted in Figure 16a,b, it can be seen that the statistics level are different between Figure 16a and Figure 16b. Subsequently, the detection results that maintain the same number of false alarms are illustrated in Figure 16c,d. With three false alarms, we can see that all targets are detected in Figure 16d, however, target 3 and 5 with lower SCR are not detected in Figure 16c. The main reason is that PCA can preserve the main components in the range cell that represent the target and reduce the redundant information, then the SCR is improved and the target is highlighted, so that the distinguishability between target and clutter can be enhanced. Therefore, by performing the proposed PCA method, the target can be better detected in the environment with lower SCR.

## 6. Conclusions

In this paper, for a smaller bunch of pulses, a PCA-based matrix CFAR detection method, which takes advantage of the superiority of geometry detection, was proposed for radar target detection. Concretely, the PCA method was carried out to reduce the dimensionality and redundant information of Toeplitz HPD covariance matrix, and the SCR was improved by capturing the main information. Subsequently, two Toeplitz HPD covariance matrix estimation algorithms that are empirical mean (EM) algorithm and regularized Burg (RB) algorithm have been considered. By performing the proposed PCA method, the detection performance has been improved via simulation experiment and real sea clutter experiment. In addition, the numerical experimental results have shown that the proposed PCA method outperforms original matrix CFAR detection method and classical FFT-CFAR detection method. Meanwhile, the detection performance of PCA method under two estimation algorithms are superior to Median Burg method, and the better performance has been validated with respect to the cases of different scenarios and extended target. Future work may focus on the concrete investigation on why the detection performance with RD improves more significantly than KLD after PCA.

## Figures and Tables

**Table 1:** Procedure of PCA-Based Matrix CFAR Detection Method.

Method Steps
Step 1: estimate the HPD matrix of each cell by the received observation data,
Step 2: apply PCA to covariance matrix $R_{D}$ of the cell under test, obtain mapping matrix V and dimensionality
reduced matrices $R_{D}^{↓}$, $R^{↓}$,
Step 3: compute geometric mean ${\bar{R}}^{↓}$ and geometric distance $d(R_{D}^{↓},{\bar{R}}^{↓})$,
Step 4: make detection decision $d\left(R_{D}^{↓},{\bar{R}}^{↓}\right)\overset{target}{\underset{no\;target}{≷}}\gamma$.

**Table 2:** Parameters of Point Targets.

Target Name	Location of Range Cell	SCR	Doppler Frequency
target 1	3th	−5 dB	−401.4 Hz
target 2	3th	0 dB	98.6 Hz
target 3	9th	−17 dB	−287.1 Hz
target 4	9th	5 dB	−44.3 Hz
target 5	13th	−15 dB	27.1 Hz
